# Experimental study of EUV mirror radiation damage resistance under long-term free-electron laser exposures below the single-shot damage threshold

**DOI:** 10.1107/S1600577517017362

**Published:** 2018-01-01

**Authors:** Igor A. Makhotkin, Ryszard Sobierajski, Jaromir Chalupský, Kai Tiedtke, Gosse de Vries, Michael Störmer, Frank Scholze, Frank Siewert, Robbert W. E. van de Kruijs, Igor Milov, Eric Louis, Iwanna Jacyna, Marek Jurek, Dorota Klinger, Laurent Nittler, Yevgen Syryanyy, Libor Juha, Věra Hájková, Vojtěch Vozda, Tomáš Burian, Karel Saksl, Bart Faatz, Barbara Keitel, Elke Plönjes, Siegfried Schreiber, Sven Toleikis, Rolf Loch, Martin Hermann, Sebastian Strobel, Han-Kwang Nienhuys, Grzegorz Gwalt, Tobias Mey, Hartmut Enkisch

**Affiliations:** a Industrial Focus Group XUV Optics, MESA+ Institute for Nanotechnology, University of Twente, Drienerlolaan 5, 7522 NB Enschede, The Netherlands; b Institute of Physics, Polish Academy of Sciences, Al. Lotników 32/46, PL-02-668 Warsaw, Poland; c Institute of Physics, Academy of Sciences of the Czech Republic, Na Slovance 2, 182 21 Prague 8, Czech Republic; d Deutsches Elektronen-Synchrotron DESY, Notkestrasse 85, Hamburg 22607, Germany; e ASML Netherlands BV, PO Box 324, Veldhoven, 5500 AH, The Netherlands; f Helmholtz-Zentrum Geesthacht, Max-Planck-Strasse 1, Geesthacht 21502, Germany; g Physikalisch-Technische Bundesanstalt, Abbestrasse 2-12, Berlin 10587, Germany; h Helmholtz Zentrum Berlin für Materialien und Energie, Albert-Einstein-Strasse 15, Berlin 12489, Germany; i Institute of Plasma Physics, Academy of Sciences of the Czech Republic, Za Slovankou 3, 182 00 Prague 8, Czech Republic; j MFF, Institute of Physics of Charles University, Ke Karlovu 5, Prague 2, Czech Republic; k Institute of Materials Research, Slovak Academy of Sciences, Watsonova 47, Košice 040 01, Slovak Republic; l Carl Zeiss SMT GmbH, Rudolf-Eber-Strasse 2, Oberkochen 73447, Germany; m Laser-Laboratorium Göttingen eV, Hans-Adolf-Krebs-Weg 1, Göttingen 37077, Germany

**Keywords:** free-electron laser induced damage, EUV optics, thin films, FELs

## Abstract

The durability of grazing- and normal-incidence optical coatings has been experimentally assessed under free-electron laser irradiation at various numbers of pulses up to 16 million shots and various fluence levels below 10% of the single-shot damage threshold. The experiment was performed at FLASH, the Free-electron LASer in Hamburg, using 13.5 nm extreme UV (EUV) radiation with 100 fs pulse duration. Polycrystalline ruthenium and amorphous carbon 50 nm thin films on silicon substrates were tested at total external reflection angles of 20° and 10° grazing incidence, respectively. Mo/Si periodical multilayer structures were tested in the Bragg reflection condition at 16° off-normal angle of incidence. The exposed areas were analysed post-mortem using differential contrast visible light microscopy, EUV reflectivity mapping and scanning X-ray photoelectron spectroscopy. The analysis revealed that Ru and Mo/Si coatings exposed to the highest dose and fluence level show a few per cent drop in their EUV reflectivity, which is explained by EUV-induced oxidation of the surface.

## Abstract 2

An experimental study of the durability of extreme UV optical coatings to a large number of free-electron laser pulses is reported.

## 1. Introduction   

The intense radiation of free-electron lasers (FELs) can damage the optical coating in a single shot if the power exceeds the so-called single-shot damage threshold (SSDT) (Khorsand *et al.*, 2010 ▸). At a pulse length of 100 fs and shorter, the phonon system, that typically reacts on a ps time scale, is too slow to carry away energy from the irradiated volume during the pulse. Therefore it is not the peak power of the pulse that determines the damage threshold but the total energy per pulse per unit of area. This is called the fluence of a pulse. The knowledge of the SSDT value gives only an estimate of the highest power that can be reflected without permanent damage to the coating. In the case of a large number of pulses with a fluence below the SSDT, damage accumulation can take place similar to heat accumulation as reported by Sobierajski *et al.* (2016 ▸). Moreover, heat accumulation is not the only possible cause of damage. The high-power ultra-short pulses deliver enough energy to the system to cause re-crystallization and an increase in the number of defects in thin films, that eventually will lead to optics damage (Mannion *et al.*, 2004 ▸). Since a FEL was proposed as a source for EUV photolithography, an application where the optics is subject to a large number of pulses (Müller *et al.*, 2012 ▸) at high repetition rate, high power and long-term operation, a rigorous study of the multi-shot damage threshold is required. This paper describes an experimental evaluation of the durability, or stability, of grazing- and normal-incidence coatings optimized for 13.5 nm wavelength to long-term FEL irradiation.

We have studied changes in optical coatings induced by a large number of pulses at various fluence levels below the SSDT. Three of the most interesting materials for extreme UV (EUV) radiation optics were tested, namely ruthenium and carbon coatings as grazing-incidence mirrors and a periodic Mo/Si multilayer as a near-normal incidence mirror. Carbon coatings are currently used in the Free-electron LASer in Hamburg (FLASH) optical path and show good performance. Ruthenium is commonly used as a protecting capping layer for EUV multilayer mirrors as it has good chemical and radiation stability and a high critical angle of total reflection for EUV. Mo/Si multilayers are the basis for reflective coatings used in EUV photolithography (Louis *et al.*, 2011 ▸).

The experiments have been executed at FLASH (Tiedtke *et al.*, 2009 ▸; Ackermann *et al.*, 2007 ▸). The exposures were performed for various fluences below 10% of the SSDT values for all three materials and for various numbers of pulses up to 16 million. The long-term FEL-irradiated spots were characterized using EUV reflectivity and X-ray photoelectron spectroscopy (XPS).

## 2. Experiment description   

The FEL was tuned to 13.5 nm wavelength. The samples were mounted such that they deflected the beam horizontally, resulting in a *p*-polarized reflection geometry. In the experiment two illumination regimes were used, namely single-shot and multi-shot. In the single-shot mode, the FLASH generates single pulses with a repetition rate of 10 Hz. In the multi-shot mode, FLASH generates pulse trains that contain 400 shots with 1 µs time separation resulting in pulse trains with a duration of 399 µs. Again, the pulse trains are generated with the repetition rate of 10 Hz. A fast shutter allows one to separate pulses in the 10 Hz time frame and thus to select any number of single pulses in the single-shot mode and, respectively, pulse trains of 400 pulses in the multi-shot regime (Tiedtke *et al.*, 2009 ▸). A combination of a gas cell and metallic filter foils is used to attenuate the energy per pulse in the beam. The FEL beam was focused to the position of the experimental chamber using a carbon-coated elliptical focusing mirror working at 3° grazing incidence. The major part of the irradiations was done on samples located out of the focus of the focusing mirror to have a spot diameter of about 0.5 mm enabling EUV reflectometry and XPS mapping.

The FELIS experimental chamber, described by Sobierajski *et al.* (2013 ▸), that was especially designed for optics damage studies, was used. The chamber was vented every time a sample was changed, and could not be baked to avoid possible damage to the coatings. The vacuum was kept below 10^−6^ mbar (10^−4^ Pa). To expose several spots on each sample, the samples were moved under vacuum, the movement being monitored by an in-line microscope. The pulse intensity on the sample was controlled by both attenuation with gas and thin solid film attenuators, and motorized movement of the entire chamber with respect to the focus position along the beam path.

The samples were prepared by magnetron sputtering in an Ar atmosphere. Thin films were coated on super-polished Si substrates of <2 Å root mean square roughness, as measured by atomic force microscopy (AFM). No increase in the roughness was found after coating. The coating thickness is 50 nm for both Ru and C, designed for grazing-incidence reflection below the critical angle, where the EUV penetration depth is in the order of 5 nm. Therefore, a 50 nm coating can be considered thick enough to absorb all EUV radiation. The Mo/Si periodic coating contained 50 bilayers with an individual bilayer thickness of 7.2 nm. Thus the Bragg reflection condition occurs at 16.26° off-normal for *p*-polarized light at 13.5 nm.

## 3. Beam characterization and determination of the single-shot damage threshold values   

The preparation for the irradiation phase consisted of the following actions that are explained below in the text. (i) Finding the focal position; (ii) characterization of the beam size in focus; (iii) study of the SSDTs for coatings under investigation; (iv) changing the position of the chamber to the out-of-focus condition; (v) beam characterization out of focus.

The focal spot was found using a so-called *z*-scan procedure (Chalupský *et al.*, 2011 ▸). During the *z*-scan a 5 µm-thick photosensitive layer of polymethyl­methacrylate (PMMA) is exposed to a FEL beam at various *z* positions along the beam path. After exposure, the areas of the ablated craters at these positions were measured using visible light microscopy with differential interference contrast (DIC or Nomarski). The *z* position where the smallest spot was observed was selected as the focal position. For long-term irradiation below the SSDT the largest possible spot that still has enough fluence to reach close to 10% of the SSDT for all coatings was selected. For SSDT determination the highest possible flux density was preferred. Therefore, two different positions of the exposure chamber along the beam axis were used, namely the in-focus position for the high-intensity regime and the out-of-focus position for the low-intensity regime. Experiments carried out in the focal spot are marked InF.

**Figure 1:**
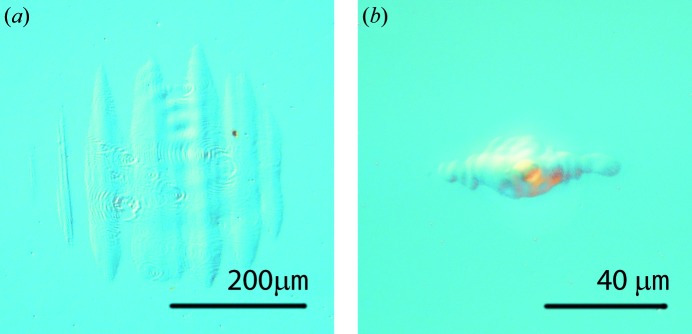
Out-of-focus (*a*) and in-focus (*b*) ablation imprints in PMMA.

The effective areas of the FEL laser beam were determined for in- and out-of-focus chamber positions by the fluence scan technique (f-scan), that involves the analysis of the dependence of ablation imprint areas on the FEL pulse energy (Chalupský *et al.*, 2010 ▸). Since the peak fluence at the out-of-focus position is significantly reduced, an alternative method of desorption imprints (Chalupský, Juha *et al.*, 2009 ▸) was used for more precise characterization. The effective area was defined as 64 100 µm^2^ for the out-of-focus position and 41 µm^2^ for the in-focus position. These values of effective areas *A*_eff_ determine the relation *F*_0_ = *E*/*A*_eff_, where *E* is the energy of the beam and *F*_0_ is the peak fluence. DIC microscopy images of in- and out-of-focus beam ablation imprints are shown in Fig. 1 ▸.

A procedure similar to the f-scan method was used in the experimental determination of the SSDT for optical coatings. In our experiment the SSDT is defined as the maximum pulse energy that does not cause damage of the coating surface detectable by DIC microscopy, *i.e.* when the area of the ablation imprint becomes zero. To find this energy value Liu’s method was used (Liu, 1982 ▸).

**Figure 2:**
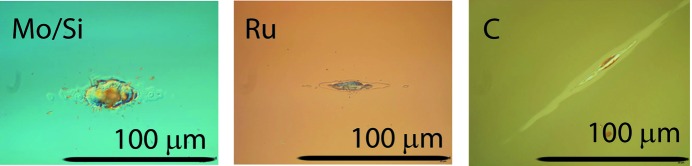
Examples of damage craters in Mo/Si, Ru and C optical coatings, recorded for fluences above the damage threshold values shown in Table 1 ▸, namely 100 J cm^−2^ for Mo/Si, 21.8 J cm^−2^ for Ru and 4.0 J cm^−2^ for amorphous C.

DIC microscopy images of the spots irradiated with FEL pulses above the damage threshold on Mo/Si (exposed at φ = 74.5° grazing angle), ruthenium (exposed at φ = 20° grazing angle) and amorphous carbon (exposed at φ = 10° grazing angle) are shown in Fig. 2 ▸. As the grazing angle drops, the length of the imprints increases in the direction of the incidence plane which is a consequence of the 1/sin(φ) scaling rule. Table 1 ▸ summarizes the SSDT levels determined during the experiment. The results are in reasonable agreement with previously reported SSDT values of 45 mJ cm^−2^ for normal-incidence reflective Mo/Si multilayer coatings (Khorsand *et al.*, 2010 ▸) and 80 mJ cm^−2^ for 4° grazing-incidence amorphous carbon (Chalupský, Hájková *et al.*, 2009 ▸). Note that the SSDT values described here were determined after the experimental campaign. However, in the first analysis during the experiments we could only roughly determine the SSDT and as a result the multiple-shot exposures with maximum fluence were carried out below the 10% of the correct SSDT value, so somewhat lower than intended. This however does not affect the following analysis.

## 4. Multi-shot exposures   

**Figure 3:**
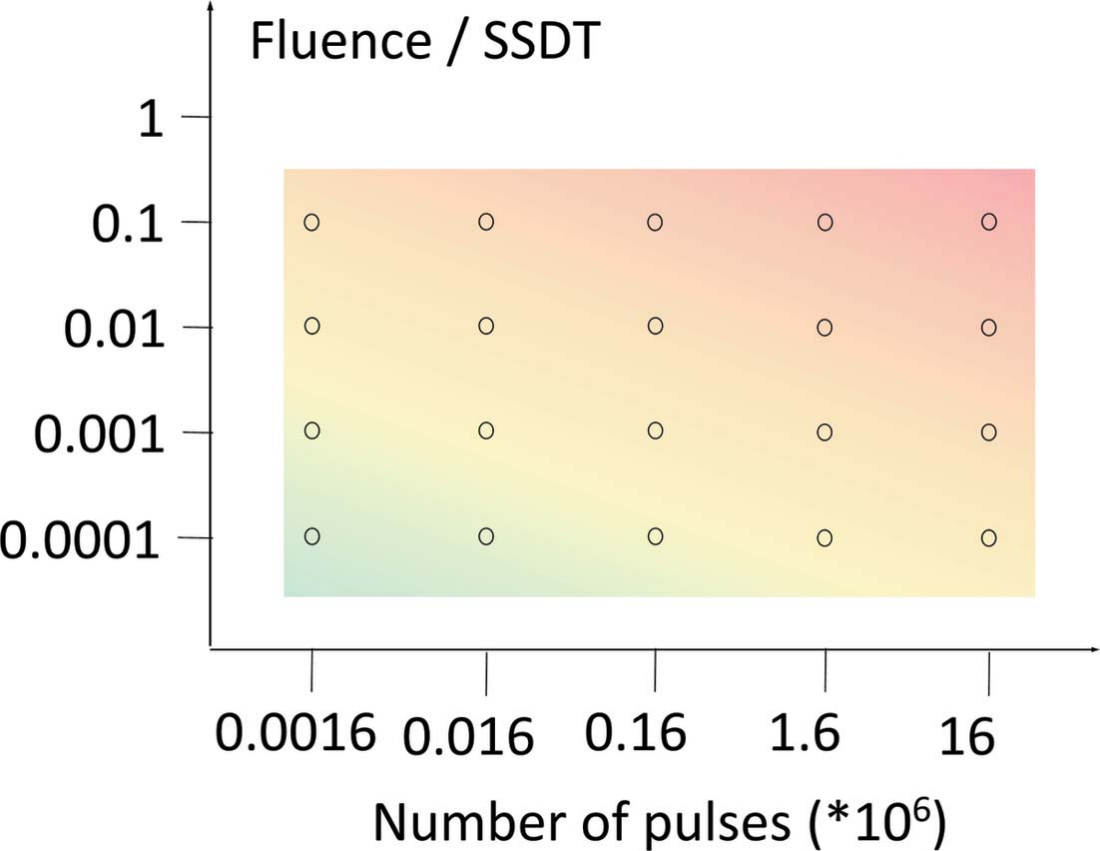
Overview of the exposure conditions in the fluence/number of pulses space.

Each coating has been exposed to various numbers of pulses at various fluence levels below the SSDT spanning a rectangle in the fluence/pulses space according to the irradiation map shown in Fig. 3 ▸. It was expected that the largest damage would occur at the highest fluence and at the largest number of pulses (top right corner in the diagram), getting lower with both the fluence and the number of pulses. The fluence levels have been calculated based on the determined damage threshold values shown in Table 1 ▸ and controlled by the transmission of the gas attenuator as well as by inserting solid absorbers into the beam (Tiedtke *et al.*, 2009 ▸).

## 5. Post-mortem analysis of the exposed spots   

During the experiment we measured the DIC microscopy images of exposed spots *ex situ*. The microscopy revealed that no craters were formed and only in spots exposed to the highest doses was a slight colouring detected. The next step in post-mortem analysis of exposed spots was EUV reflectance mapping since EUV reflectivity is the property of interest and it is a good indicator of possible changes of the structure and/or surface of the optical coatings.

**Figure 4:**
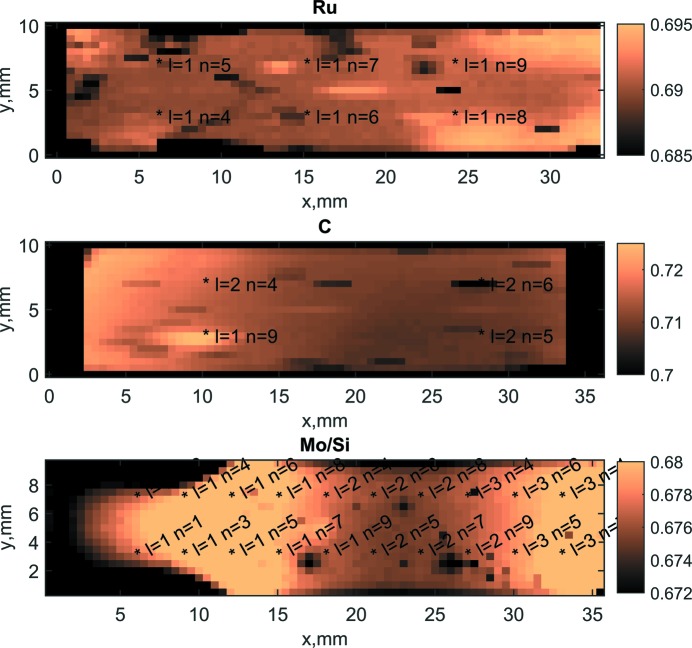
Coarse reflectivity maps of the samples exposed to the highest doses; the colour scale bars show EUV reflectivity.

The prime goal of EUV mapping was to detect if exposure to intense multiple EUV pulses induced any reflectance loss and to give indications for potential changes in the structure of the thin films and to determine the next analysis steps. The measurement of the EUV reflectivity was performed at the Physikalisch-Technische Bundesanstalt (PTB) EUV beamline at the Metrology Light Source storage ring (Laubis *et al.*, 2013 ▸). The EUV beamline uses a plane-grating monochromator with a collimated beam. The resolving power in the EUV spectral range is about 10^3^. Examples of overview maps are presented in Fig. 4 ▸.

For each coating the reflectivity was measured at a fixed wavelength of 13.5 nm and a fixed incidence angle using a spot size of 1 × 1 mm, providing an overview of the regions where the coating was possibly modified by the FEL exposure. In all maps shown in Fig. 4 ▸ we show the FEL-irradiated spots that were indicated according to the data available from the FEL irradiation scheme. The FEL irradiation type is indicated according to Tables 2 ▸ and 3 ▸. Because of the different incidence angles for Ru, C and Mo/Si the illuminated spots have different sizes. Therefore the FEL irradiations have been performed with different steps in-between to make sure that the distance between these spots has always been three times the beam spot size, in order to exclude overlapping effects.

**Figure 5:**
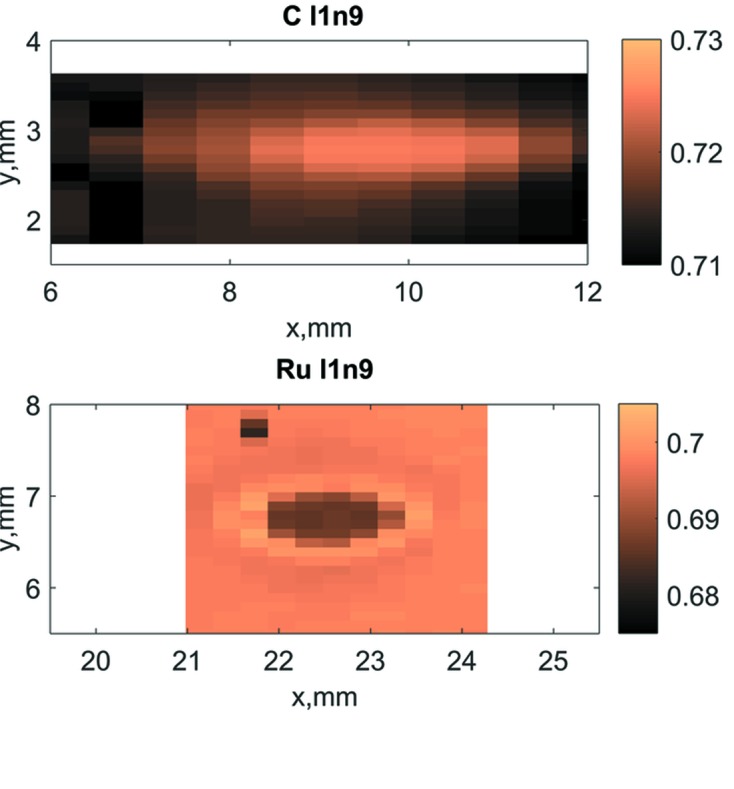
Detailed reflectivity maps for C and Ru l1n9 exposures; the colour scale bars show the EUV reflectivity scale.

In all reflectivity maps a change of reflectivity at the spot with the highest FEL irradiation dose was found. The spots that received the highest exposure were labelled l1n9, which means that according to Tables 2 ▸ and 3 ▸ these spots received 16 million shots at a fluence level close to 10% of the SSDT. For this highest exposure dose, Mo/Si and Ru coatings show a reflectivity decrease, while for the C coating the reflectivity increased. As can be seen from the reflectivity maps the real positions of FEL-irradiated spots are slightly shifted with respect to the planned positions. This can be due to a shift in the alignment of the samples to the beam. Detailed reflectivity maps in points of interest were recorded using a smallest achievable spot of the PTB reflectometer beam of 0.2 × 0.2 mm. Examples of such detailed maps for Ru and C coatings are shown in Fig. 5 ▸.

**Figure 6:**
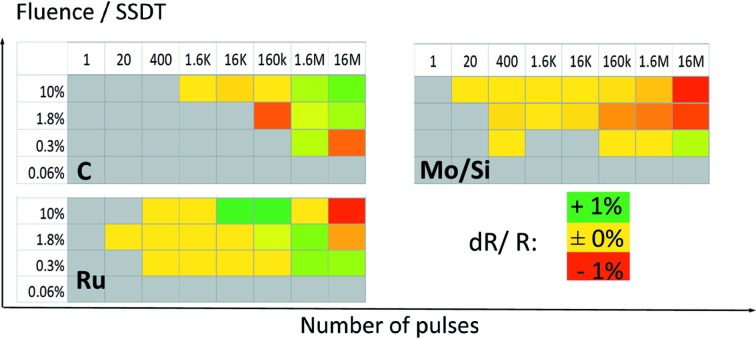
Overview of reflectivity changes detected from EUV reflectivity maps.

The reflectivity changes were determined as (*R*_E_ − *R*_BG_)/*R*_BG_, where *R*_BG_ is the background reflectivity outside the exposed spot and *R*_E_ is the reflectivity inside the exposed spot. An overview of measured reflectivity changes is given in Fig. 6 ▸, showing that all EUV reflectivity changes are in the order of 1–2%; therefore only minor structural changes are suggested. For Mo/Si only a reflectivity decrease is detected. A more complicated picture is observed for the ruthenium coating, where higher-dose irradiations cause a reflectivity decrease while lower-dose irradiations result in a slight reflectivity increase.

Several EUV-induced changes can happen to optics, starting from thermal damage [discussed for example by Sobierajski *et al.* (2011 ▸), Rost *et al.* (2003 ▸)], that causes compaction of a periodic Mo/Si multilayer (Khorsand *et al.*, 2010 ▸), to ablation of the surface (Aquila *et al.*, 2015 ▸) or EUV-induced oxidation or carbon growth (Hill *et al.*, 2007 ▸, 2008 ▸). The first two processes mentioned will cause a large reflectivity decrease. An increase of reflectivity may be caused by EUV photo-desorption or EUV-induced cleaning of C contamination as suggested by Hill *et al.* (2007 ▸, 2008 ▸).

**Figure 7:**
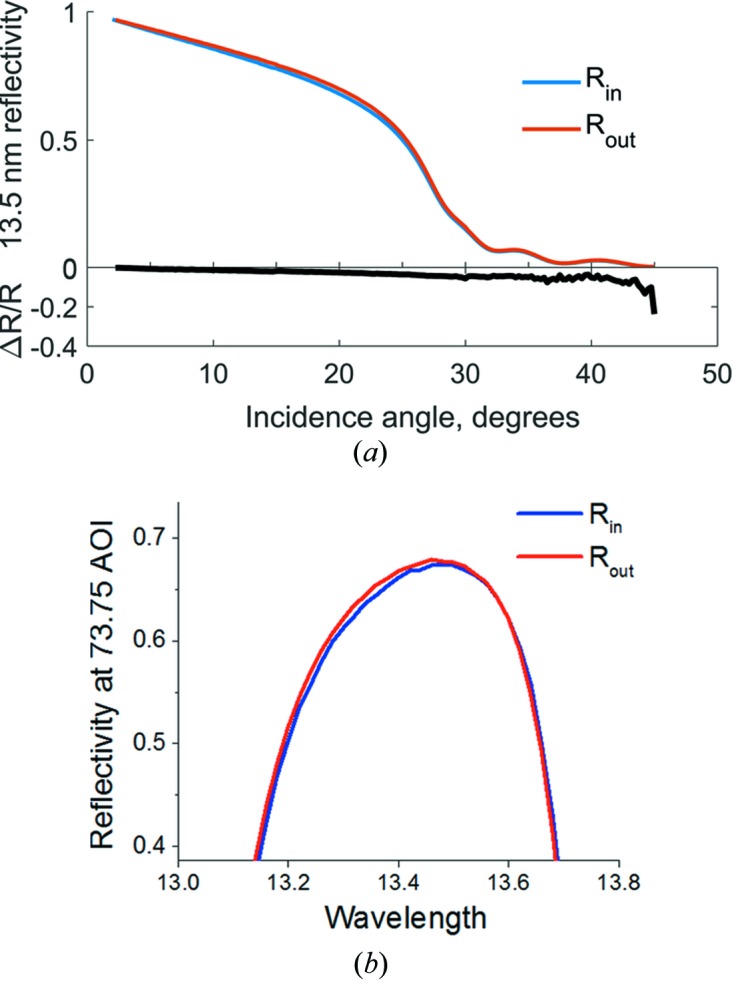
Detailed EUV reflectivity scans inside and outside the l1n9 spots: (*a*) for Ru an angle of incidence scan at fixed wavelength of 13.5 nm and (*b*) for Mo/Si a wavelength scan at a fixed angle of incidence of 73.75°.

To detect possible structural changes, reflectivity spectra have been measured inside and outside the l1n9 spots. For C and Ru we have performed angle scans at a fixed wavelength. In Fig. 7 ▸(*a*) the measurements for Ru are shown. The most prominent feature is the slight reflectance loss at angles around 10 to 20°. This loss can be explained by the increase in surface roughness by 0.8 nm, but it can also be explained by the presence of an oxide layer on the surface. Fitting of the EUV reflectance data shows that the observed changes can be explained by the increase of RuO oxide thickness by 0.5 nm. Thus, the EUV reflectance data are not enough to allow a unique conclusion on the exact cause of the small decrease of reflectivity. Unfortunately we could also not determine uniquely the causes of the change of reflectance of the C coatings.

On the Mo/Si multilayer coatings we have measured the reflectance as a function of the wavelength at a fixed incidence angle to check for possible changes in the multilayer structure. The difference of the EUV peak reflectivity for Mo/Si shown in Fig. 7 ▸(*b*) can be explained by the presence of 1.5 nm SiO_2_ on the top of the multilayer. Additionally we observe a shift of the central wavelength of the Bragg peak by 0.002 nm to lower values. This could be caused by structural changes at the Mo-on-Si and Si-on-Mo interfaces on which, in case of damage, Mo and Si form a Mo–silicide compound that leads to compaction of the period thickness and thus to a shift to smaller wavelengths. However, the extremely small change of 0.002 nm in the central wavelength of the Bragg peak is in agreement with the coating inhomogeneity, *i.e.* a small change in the as-deposited multilayer (ML) period between the spots compared. The observed slight reduction of the reflectivity (Fig. 7 ▸) is therefore most likely caused by a modification of the top surface of the mirror. Note that the surface oxidation alone also induces an asymmetric change of the peak shape which might also explain a minor shift in the centre wavelength without any change of the inner ML structure. Therefore there is no indication of interface damage and we conclude that there is no measurable change in the multilayer structure.

Despite their high sensitivity to the structural changes, EUV reflectivity data are not sufficient to draw conclusions on the nature of the changes of the structure of the analysed samples (Yakunin *et al.*, 2014 ▸; Haase *et al.*, 2016 ▸). Therefore, additional characterization techniques are needed for combined analysis. To test the hypotheses of surface contamination, XPS mapping of the spots exposed to the highest dose (l1n9) was performed. The characterization was complicated by the relatively small size of the spots to be analysed, which were 1 × 3 mm for the Ru, 1 × 6 mm for the C coatings and 1 × 1 mm for Mo/Si, that had to be found on 10 × 36 mm samples. We chose this non-destructive characterization technique to be able to re-analyse irradiated spots should it be required.

**Figure 8:**
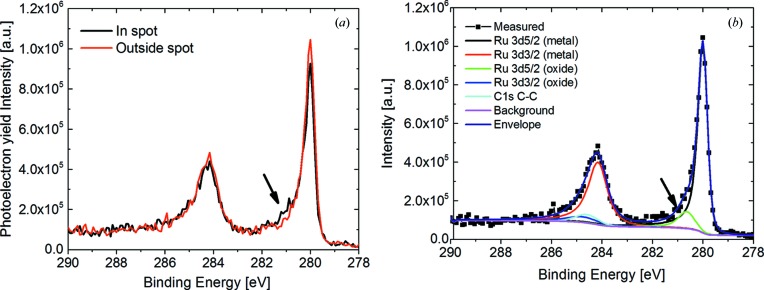
XPS spectra of Ru inside and outside the exposed (l1n9) spot (*a*) and combined with the fitting results inside the exposed spot (*b*).

**Figure 9:**
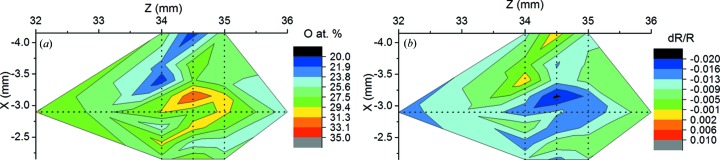
Oxygen content in the surface layer of the Ru film, determined by XPS mapping (*a*) and the simulated effect of this oxygen amount on the EUV reflectivity (*b*). The latter can be compared with EUV reflectivity shown in Fig. 5 ▸. The uncertainties of measured atomic concentrations are smaller than their spatial variations over the unirradiated area.

The XPS measurements have been performed using monochromatic Al *K*α radiation as the excitation wavelength and a 0.1 × 0.3 mm observation spot size. The XPS spectra inside and outside the exposed Ru l1n9 spot together with the fitting to the contributing electronic states are shown in Fig. 8 ▸. A minor enhancement of the XPS spectrum at 280.6 eV binding energy can be explained by the increased presence of ruthenium oxide, namely 30% in the exposed spot compared with 15% outside [green spectrum from Fig. 8 ▸(*b*)]. Such an increase would result in a decrease of the EUV reflectivity of Ru by 1%. A comparison of the oxygen-containing map from XPS and the simulated EUV reflectivity from the structure consisting of 46.8 nm Ru with 2.5 nm of Ru_1−*x*_O_*x*_ on top, where *x* is the oxygen content from XPS analysis, is shown in Fig. 9 ▸. Unfortunately, because of the overlap of the C 1*s* and Ru 3*d* features in the XPS spectra we cannot draw any conclusion on whether there is an increase or a decrease of the C content on the surface.

Nevertheless, combining the XPS measurements that show an increase of the oxygen content in the exposed spots with EUV reflectivity measurements that show a reflectance drop on an area of the same size, we conclude that the EUV-induced oxidation is indeed the main cause of the observed reduced EUV reflectivity.

The XPS analysis of the l1n9 spot on the carbon sample did not reveal detectable changes of the surface chemistry.

**Figure 10:**
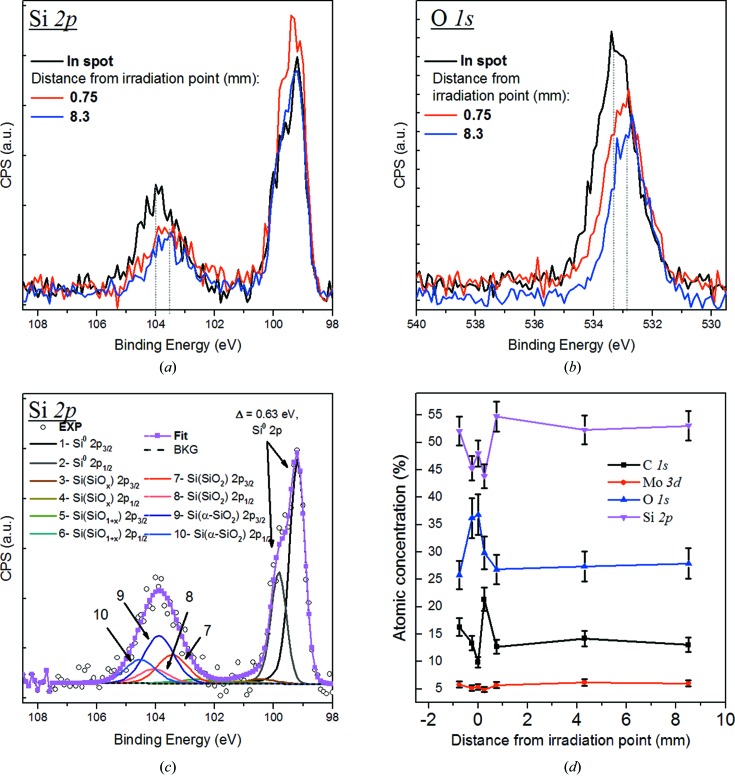
XPS data inside and outside the Mo/Si l1n9 spot: (*a*) and (*b*) show the Si 2*p* and O 1*s* spectra measured in the spot position and at the distance of 0.75 and 8.3 mm from the centre of the irradiated spot. (*c*) shows the decomposition of Si 2*p* spectra, and (*d*) the atomic concentration of the elements *versus* distance from the irradiation centre.

A similar analysis was performed for the Mo/Si multilayer surface; this confirmed an increased oxygen content of the multilayer surface as well as a slight increase of the carbon content (see Fig. 10 ▸). Also, here, the observed increase of the oxygen and carbon on the surface gives a strong indication that under the described exposure conditions EUV-induced surface contamination is the prime cause of the observed EUV reflectivity changes of the Mo/Si multilayer structures.

## 6. Conclusions   

The analysis performed shows that FEL exposures at pulse energies below the SSDT level affect the top surface of Ru, C and Mo/Si optical coatings, influencing their EUV reflectance. The spots irradiated by 16 million pulses with an energy per pulse below 10% of the SSDTs for the corresponding materials were analysed in detail. Angular- and wavelength-dependent EUV reflectivity analysis did not reveal changes of the internal structure of the optical coatings. This result indicates that even if long-term pulsed EUV irradiation would cause accumulation of irreversible damage, 16 million pulses are not enough to create detectable changes. The XPS analysis shows that the reduction of reflectivity for grazing-incidence ruthenium and near-normal incidence Mo/Si mirrors exposed can be attributed to the formation of EUV-induced surface oxide. These effects can be minimized by improving the vacuum condition in the optical chamber or applying optics-cleaning procedures.

## Figures and Tables

**Table 1:** Summary of single-shot damage analysis results. Note that the SSDT energy in the beam for Mo/Si is less than for amorphous C but on the sample it is the other way around, which is a consequence of the enlarged footprint at grazing angles of incidence

Material	Threshold energy in the beam *E*_th_ (µJ)	Grazing angle φ (°)	Effective area of the beam *A*_eff_ (µm^2^)	Effective area on the sample *A*_eff_/sin(φ) (µm^2^)	Threshold fluence on the sample *F*_th_ (mJ cm^−2^)
Mo/Si (InF)	0.03	74.5	41	66.6	83
Ru (InF)	0.2	20	41	187.6	199
Amorphous C (InF)	0.046	10	41	369.5	24

**Table 2:** List of attenuation levels used for exposures with notations (for example, l3 denotes 0.33% of the SSDT)

l index	1	2	3	4	5	6
Exposure level in % of SSDT	10	1.8	0.33	0.06	0.011	0.002

**Table 3:** List of exposure types and their notations (for example, n5 denotes 4 shots with 400 pulses in each shot and a total of 1600 pulses)

Exposure type, n	Pulses in a shot	*N* shots	Total pulses
1	1	1	1.0E+00
2	1	20	2.0E+01
3	1	400	4.0E+02
4	400	1	4.0E+02
5	400	4	1.6E+03
6	400	40	1.6E+04
7	400	400	1.6E+05
8	400	4000	1.6E+06
9	400	40000	1.6E+07
